# Linking Bacterial-Fungal Relationships to Microbial Diversity and Soil Nutrient Cycling

**DOI:** 10.1128/mSystems.01052-20

**Published:** 2021-03-23

**Authors:** Shuo Jiao, Ziheng Peng, Jiejun Qi, Jiamin Gao, Gehong Wei

**Affiliations:** a State Key Laboratory of Crop Stress Biology in Arid Areas, College of Life Sciences, Northwest A&F University, Yangling, Shaanxi, People’s Republic of China; b Shaanxi Key Laboratory of Agricultural and Environmental Microbiology, College of Life Sciences, Northwest A&F University, Yangling, Shaanxi, People’s Republic of China; Pacific Northwest National Laboratory

**Keywords:** neutral community assembly, ecosystem types, biodiversity-function relationships, cross-biome, nutrient cycling, negative species associations, stochastic community assembly, terrestrial ecosystems

## Abstract

Biodiversity is important for supporting ecosystem functioning. To evaluate the factors contributing to the strength of microbial diversity-function relationships in complex terrestrial ecosystems, we conducted a soil survey over different habitats, including an agricultural field, forest, wetland, grassland, and desert. Soil microbial multidiversity was estimated by the combination of bacterial and fungal diversity. Soil ecosystem functions were evaluated using a multinutrient cycling index (MNC) in relation to carbon, nitrate, phosphorus, and potassium cycling. Significant positive relationships between soil multidiversity and multinutrient cycling were observed in all habitats, except the grassland and desert. Specifically, community compositions showed stronger correlations with multinutrient cycling than α-diversity, indicating the crucial role of microbial community composition differences on soil nutrient cycling. Importantly, we revealed that changes in both the neutral processes (Sloan neutral modeling) and the proportion of negative bacterial-fungal associations were linked to the magnitude and direction of the diversity-MNC relationships. The habitats less governed by neutral processes and dominated by negative bacterial-fungal associations exhibited stronger negative microbial *α*-diversity–MNC relationships. Our findings suggested that the balance between positive and negative bacterial-fungal associations was connected to the link between soil biodiversity and ecosystem function in complex terrestrial ecosystems. This study elucidates the potential factors influencing diversity-function relationships, thereby enabling future studies to forecast the effects of belowground biodiversity on ecosystem function.

**IMPORTANCE** The relationships between soil biodiversity and ecosystem functions are an important yet poorly understood topic in microbial ecology. This study presents an exploratory effort to gain predictive understanding of the factors driving the relationships between microbial diversity and potential soil nutrient cycling in complex terrestrial ecosystems. Our structural equation modeling and random forest analysis revealed that the balance between positive and negative bacterial-fungal associations was clearly linked to the strength of the relationships between soil microbial diversity and multiple nutrients cycling across different habitats. This study revealed the potential factors underpinning diversity-function relationships in terrestrial ecosystems and thus helps us to manage soil microbial communities for better provisioning of key ecosystem services.

## INTRODUCTION

The assembly processes and functional contributions of ecological communities are key topics in community ecology (1). In nature, community assembly processes are influenced by both deterministic (niche) and stochastic (neutral) processes, and the relative importance of these processes changes in space and time (2–4). Deterministic processes involve nonrandom and niche-based mechanisms (5), including environmental filtering and interspecific interactions (e.g., competition, facilitation, mutualisms, and predation). In contrast, stochastic processes reflect mainly random changes in the relative abundances of species, involving random birth, death, and dispersal events (6, 7). Uncovering the community assembly processes is critical to understanding the generation of biodiversity and its contribution to ecosystem functions (8–10). On one hand, stochastic assembly processes were dominant in high-α-diversity communities, associating with the existence of specialized functions that are correlated with specific bacterial taxa (9). On the other hand, the prediction of niche theory stating that competing species lead to reduced niche overlap implies a negative covariation between species through competition (11). For example, the global niche differentiation between fungi and bacteria relating to contrasting diversity responses to precipitation (12) might induce negative bacterial-fungal covariation, whereas niche partitioning may promote positive species covariations due to fitness differences among organisms under environmental heterogeneity and the niche-partitioning scenario (13, 14), which might consequently increase functional community performance (15, 16). For example, bacteria profiting from the organic matter degradation of fungi (17) may induce their positive covariation. Therefore, revealing the balance between deterministic and stochastic processes may help to predict the functional contributions of ecological communities, which should be higher in real-world environments than in simplified experimental settings (11). Given this point, it is of great importance to understand how these naturally diverse and fluctuating communities are organized (e.g., community assembly) and how they influence the functioning of ecosystems.

Biodiversity is known as a critical determinant of ecosystem functioning (18). Understanding the relationship between biodiversity and ecosystem functioning is a central issue in ecology that contributes to better provision of key ecosystem services to humans (19). The strength of the biodiversity-ecosystem function (BEF) relationship may be determined by functional redundancy, complementarity, or species competition (19–21). For example, losses of one or a few species in a community with high functional redundancy may have minimal consequences for ecosystem function (22); the complementarity effect describes that niche differentiation or facilitation between species can increase the functional performance of communities (23), and species competition for resources can reduce community functioning when species performing major functions are inhibited (20). However, these previous studies have been based on well-manipulated experimental conditions (16, 19, 20); most natural communities feature a highly complex taxonomic diversity (18, 24). Currently, it is very challenging to characterize the determinants of BEF relationships in complex natural ecosystems.

The activities of microorganisms and their interactions greatly influence a variety of ecosystem processes associating with soil productivity and nutrient cycling, as well as many other ecosystem properties and services (24). Soil bacteria and fungi may share common resources, and competition for a substrate might induce the antagonism between bacteria and fungi (25). In addition, some soil-derived fungal and bacterial species may synthesize antibiotics (26, 27), substantially affecting the species interactions. Bacteria may exhibit antifungal activity via producing volatile compounds, which were reported to inhibit the germination of fungal spores as well as hyphal growth and to change fungal morphology, enzyme activity, and gene expression (28). Species in highly competitive communities often grow less efficiently due to intense competition for shared resources (16, 20). Antagonistic interactions of Pseudomonas fluorescens communities measured *in vitro* were related to bacterial root colonization and host plant protection, suggesting that increasing antagonistic interactions may cause negative BEF relationships (20). In turn, coexisting species resulting from niche partitioning by distinct resources may positively interact, which can increase functional community performance (14). For example, soil fungi may decompose the recalcitrant organic matter, e.g., cellulose and lignin, and bacteria may symbiotically utilize fungus-derived substrates (17). Since complex soil processes are driven by interactions among soil bacteria and fungi, etc., revealing the intrinsic linkages between microbial interactions and community functioning may facilitate the management of microbial communities for improving ecosystem service provisioning (29–31).

Here, we aimed to evaluate the potential factors which affect the strength of biodiversity-function relationships in complex terrestrial ecosystems. To address this issue, we conducted a large-scale soil survey ranging over different habitats, including an agricultural field, forest, wetland, grassland, and desert, along the Hexi Corridor (transect intervals of 1,257.6 km) (see Fig. S1 in the supplemental material), which is a representative of an oasis-desert ecotone in the arid regions of northwest China (32). This specific ecological environment of oases scattered along the narrow desert belt contains various ecosystems (32). We also took advantage of the strong changes in the soil biodiversity and processes that occur vertically along the soil profile. In total, 251 soil samples at 126 sites with two soil depth layers were selected in an agricultural field, forest, wetland, grassland, and desert. This cross-habitat environmental gradient offers a model system with an ecosystem and biodiversity gradient for the investigation of the relationships between biodiversity and ecosystem function (multinutrient cycling, for example). To control the effect of spatial scale, the sampling sites for each habitat were evenly distributed along the transect of the Hexi Corridor. We hypothesized that (i) microbial diversity-ecosystem function relationships would exhibit habitat-specific patterns and would be influenced by the community assembly processes and the balance between negative and positive species associations and that (ii) more negative than positive associations between bacteria and fungi would decrease the diversity-function link, along with decreasing neutral assembly processes. Our results may help predict and regulate biodiversity-driven ecosystem functioning and further develop proper land use strategies for improving the provision of key ecosystem services.

10.1128/mSystems.01052-20.2 FIG S1 Distribution of the 126 sampling sites along the Hexi Corridor in northwest China. The sites were based in an agricultural field, forest, wetland, grassland, and desert. Download FIG S1, TIF file, 3.0 MB. Copyright © 2021 Jiao et al. 2021 Jiao et al. https://creativecommons.org/licenses/by/4.0/ This content is distributed under the terms of the Creative Commons Attribution 4.0 International license.

## RESULTS

**FIG 1:**
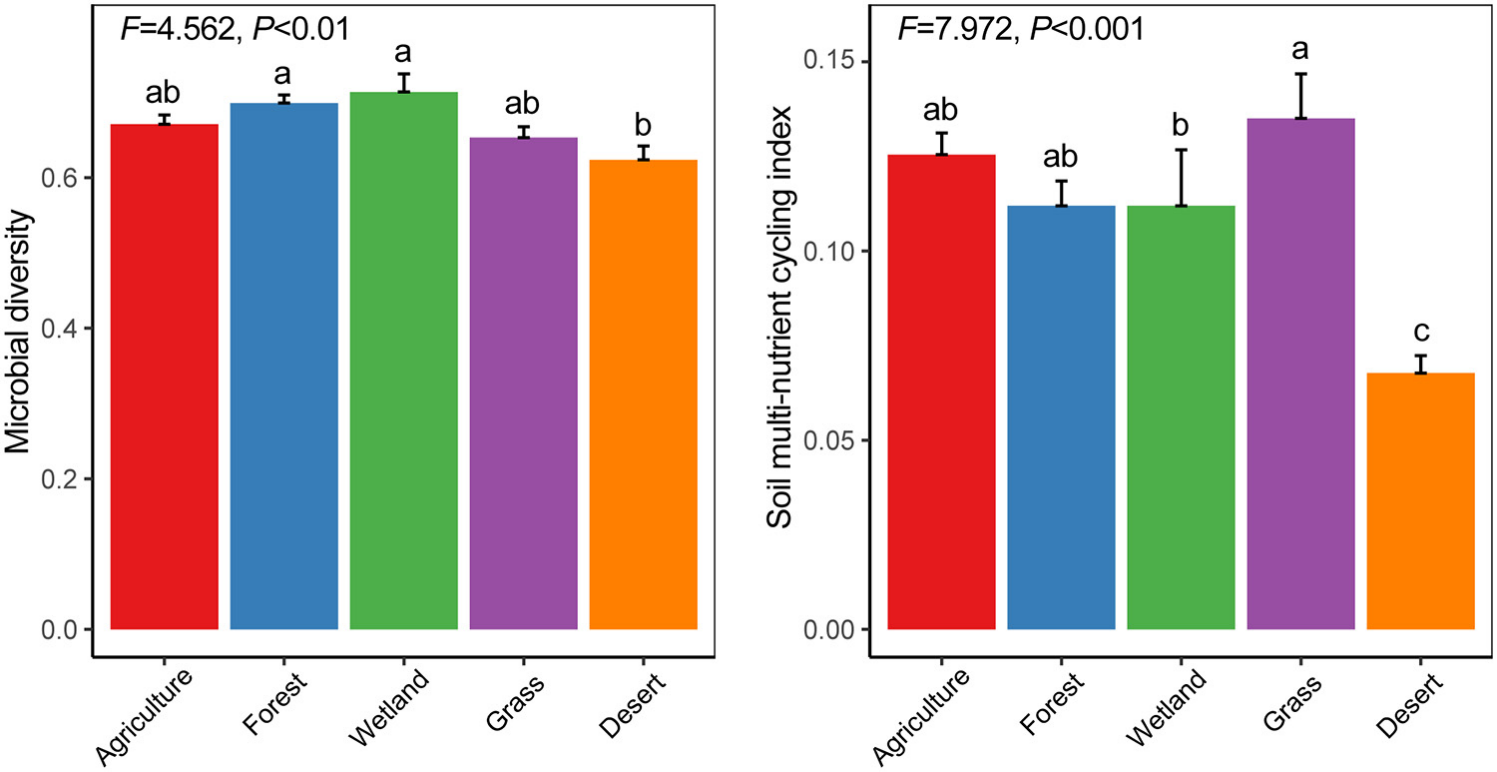
Comparison of microbial *α*-diversities and soil multinutrient cycling among different habitats. The microbial *α*-diversity was calculated as the average value of bacterial and fungal diversities after minimum-maximum normalization. The soil multinutrient cycling index (MNC) was calculated as the average value of soil organic carbon, dissolved organic carbon, microbial biomass carbon, nitrate-nitrogen, ammonium-nitrogen, microbial biomass nitrogen, available phosphorus, and available potassium contents after minimum-maximum normalization. The overall differences among habitats were estimated based on parametric one-way analysis of variance (ANOVA). In addition, different lowercase letters within panels indicate significant differences among the habitats (*P < *0.05), which were found by multiple-comparison test after Kruskal-Wallis analysis. Error bars represent the standard errors. *F*, F-statistic.

A total of 15,429,528 and 19,350,877 high-quality bacterial and fungal sequences were acquired from 251 cross-habitat soil samples along the Hexi Corridor (transect intervals of 1,257.6 km), which were, respectively, grouped into 25,981 and 21,698 operational taxonomic units (OTUs). Our results showed that the microbial *α*-diversity and multinutrient cycling index (MNC) in desert soils were lower than in other habitats (Fig. 1). The microbial *α*-diversities did not significantly differ between surface (depth, 0 to 15 cm) and subsurface (depth, 15 to 30 cm) soils in any of the habitats (see Fig. S2 in the supplemental material). In agricultural and forest soils, the MNC values were significantly higher in the surface soils than in the subsurface soils (Fig. S2). Moreover, there were significant differences in the microbial community compositions among the five habitats and between the two soil layers (Fig. S3A). In addition, a significant correlation between microbial *α*-diversities and community compositions was observed (Fig. S3B).

10.1128/mSystems.01052-20.3 FIG S2 Comparison of microbial *α*-diversity (top) and soil multinutrient cycling (bottom) values between soil layers in the different habitats. The boxplot shows differences between surface (Up, 0 to 15 cm) and subsurface (Down, 15 to 30 cm) soil samples. Blue asterisks indicate that the values were significantly higher in the surface layer (*, *P < *0.05; **, *P < *0.01, Wilcoxon rank-sum test). Download FIG S2, TIF file, 0.5 MB. Copyright © 2021 Jiao et al. 2021 Jiao et al. https://creativecommons.org/licenses/by/4.0/ This content is distributed under the terms of the Creative Commons Attribution 4.0 International license.

10.1128/mSystems.01052-20.4 FIG S3 (A) Nonmetric multidimensional scaling (NMDS) ordination plot showing the microbial community composition (calculated by combining the bacterial and fungal communities) among different habitats and between surface (Up) and subsurface (Down) soil layers. An analysis of similarities (ANOSIM) was used to determine significant differences in microbial community composition. Similarity values (*R*) of the samples among the five habitats (Habitat) and between two soil layers (Layers) were examined using the analysis of similarities and are shown at the bottom of the plot (***, *P < *0.001). (B) Relationships between microbial *α*-diversity and community composition (β-diversity). The microbial *α*-diversity was calculated as the average value of bacterial and fungal diversity after minimum-maximum normalization. The microbial community composition was estimated using the first axis of the nonmetric multidimensional scaling analysis by combining the bacterial and fungal communities. Solid and dashed lines denote the significant Pearson’s correlations. Download FIG S3, TIF file, 1.0 MB. Copyright © 2021 Jiao et al. 2021 Jiao et al. https://creativecommons.org/licenses/by/4.0/ This content is distributed under the terms of the Creative Commons Attribution 4.0 International license.

**FIG 2:**
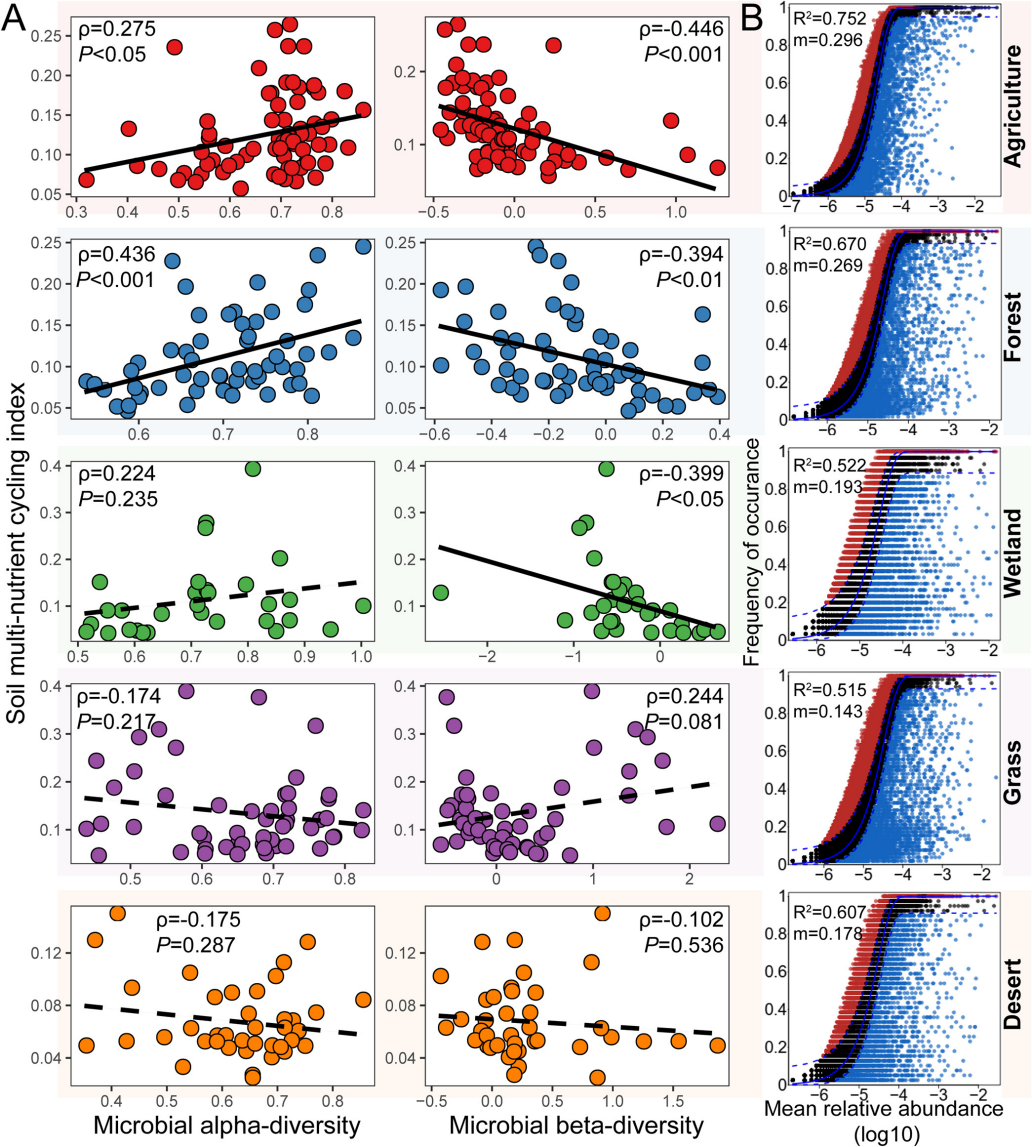
Assessment of microbial diversity-multinutrient cycling relationships and community assembly processes in agricultural field, forest, wetland, grassland, and desert soil samples from the Hexi Corridor in northwest China. (A) Relationships between microbial diversity and multinutrient cycling. The microbial *α*-diversity was calculated as the average value of bacterial and fungal diversity after minimum-maximum normalization. The microbial community composition (β-diversity) was estimated using the first axis of the nonmetric multidimensional scaling analysis by combining the bacterial and fungal communities. Solid and dashed lines, respectively, denote the significant (*P < *0.05) and nonsignificant (*P > *0.05) Pearson’s correlations. (B) Fit of Sloan’s neutral model for analysis of microbial community assembly. The analysis was based on combining the bacterial and fungal communities. The solid blue line represents the best-fitting neutral model. The dashed line represents the 95% confidence intervals (CIs) around the best-fitting neutral model. OTUs within the CIs (black points) follow the neutral process. OTUs that occur more frequently than predicted by the model are shown in red, whereas those that occur less frequently than predicted are shown in blue. *m* indicates the estimated migration rate, and *R*^2^ indicates the fit to the neutral model.

**FIG 3:**
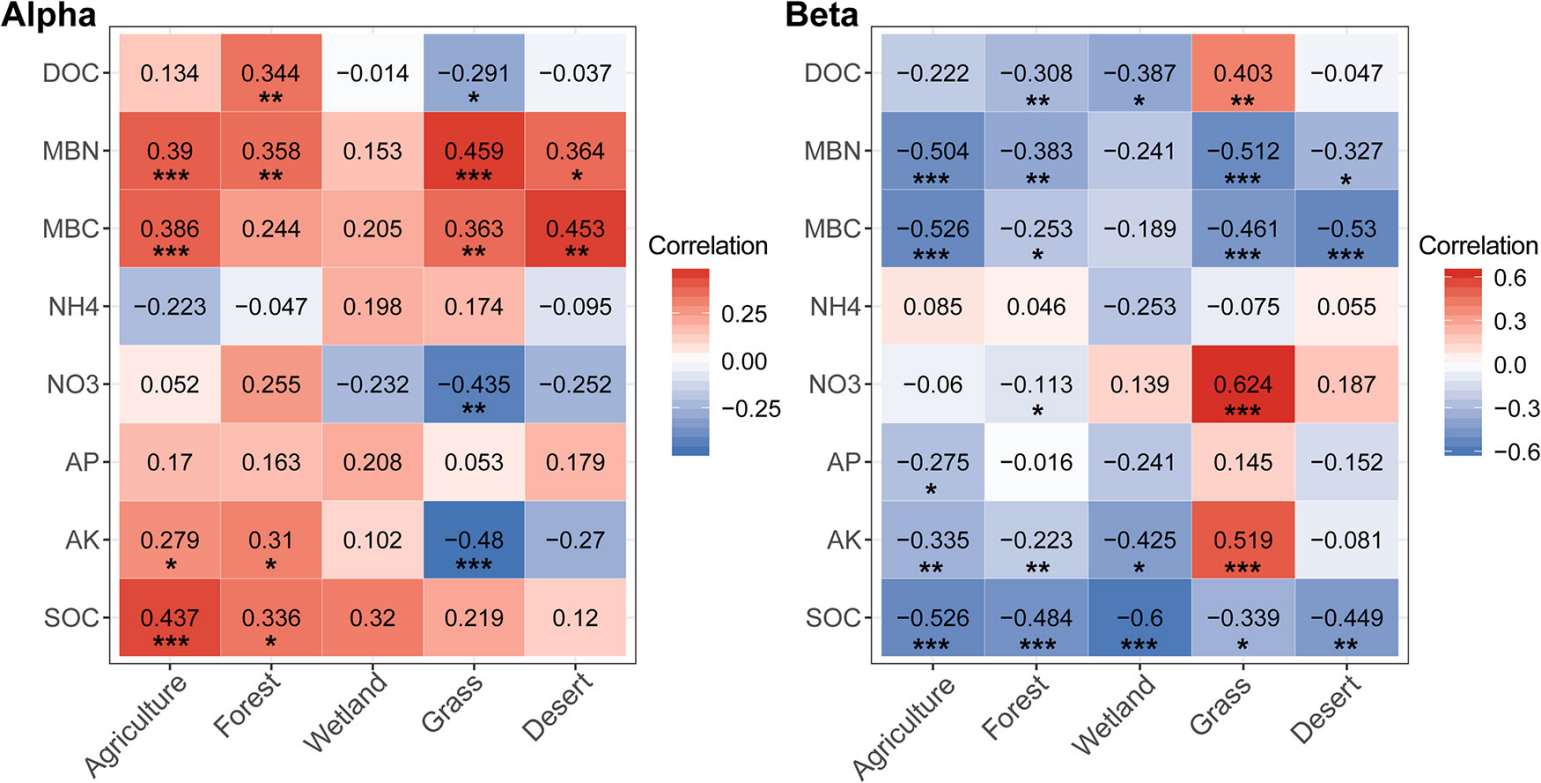
Heatmaps of correlation (Spearman’s) coefficients between microbial *α*-diversity (Alpha) and community composition (Beta) and all individual nutrient variables. The numbers in the table are *R* values. The shading from white to red represents low-to-high positive correlation, while the shading from white to blue represents low-to-high negative correlation. *, *P < *0.05; **, *P < *0.01; ***, *P* < 0.001. DOC, dissolved organic carbon; MBN, microbial biomass nitrogen; MBC, microbial biomass carbon; NH4, ammonium-nitrogen; NO3, nitrate-nitrogen; AP, available phosphorus; AK, available potassium; SOC, soil organic carbon.

The relationships between microbial diversity and the MNC were explored in the five habitats and the two soil layers. There were significant positive relationships between microbial *α*-diversity and MNC only in agricultural and forest soils (Fig. 2A). The microbial community composition was significantly correlated with the MNC in agricultural, forest, and wetland soils (Fig. 2A). Similar trends were observed in the surface and subsurface layers in different habitats (Fig. S4A and B). Concerning each component of multinutrient cycling, soil microbial diversity strongly correlated with most individual variables measured (Fig. 3). More negative correlations between microbial *α*-diversity and nutrient variables were observed in the grassland (*n *= 3) and desert (*n *= 4) than in other habitats (*n *≤ 2). The microbial community composition showed stronger correlations with most nutrient variables measured (Fig. 3) and higher correlation coefficients with MNC in 73% of habitat and soil layer combinations than *α*-diversities (Fig. 2A and Fig. S4A), indicating the major role of microbial community composition in soil nutrient cycling. This observation was further confirmed by the multiple-regression model and variation partitioning analysis (Table S1).

10.1128/mSystems.01052-20.5 FIG S4 Assessment of microbial diversity-multinutrient cycling relationships and community assembly processes in surface (Up) and subsurface (Down) layers of agricultural field, forest, wetland, grassland, and desert soils from the Hexi Corridor in northwest China. Relationships between microbial *α*-diversity (A) and community composition (β-diversity) (B) and multinutrient cycling. The microbial *α*-diversity was calculated as the average value of bacterial and fungal diversity after minimum-maximum normalization. The microbial community composition was estimated using the first axis of the nonmetric multidimensional scaling analysis by combining the bacterial and fungal communities. Solid and dashed lines, respectively, denote the significant (*P < *0.05) and nonsignificant (*P > *0.05) Pearson’s correlations. (C) Fit of Sloan’s neutral model for analysis of microbial community assembly. The analysis was based on combining the bacterial and fungal communities. The solid blue line represents the best-fitting neutral model. The dashed line represents the 95% CIs around the best-fitting neutral model. OTUs within the CIs (black points) follow the neutral process. OTUs that occur more frequently than predicted by the model are shown in red, whereas those that occur less frequently than predicted are shown in blue. *m* indicates the estimated migration rate, and *R*^2^ indicates the fit to the neutral model. Download FIG S4, TIF file, 2.6 MB. Copyright © 2021 Jiao et al. 2021 Jiao et al. https://creativecommons.org/licenses/by/4.0/ This content is distributed under the terms of the Creative Commons Attribution 4.0 International license.

10.1128/mSystems.01052-20.8 TABLE S1 Variation partitioning analysis of the relative contributions (percentages) of microbial *α*-diversity and community composition to variation in multinutrient cycling in the different habitats. Common, the simultaneous effects of *α*-diversity and community composition. Download Table S1, DOCX file, 0.04 MB. Copyright © 2021 Jiao et al. 2021 Jiao et al. https://creativecommons.org/licenses/by/4.0/ This content is distributed under the terms of the Creative Commons Attribution 4.0 International license.

We then estimated the microbial community assembly processes among the five habitats and between the two soil layers. The neutral community model explained a larger fraction of microbial community variation in agricultural soils (*R*^2^ = 0.752) than in other habitats (*R*^2^ < 0.70) (Fig. 2B). In addition, the degree of fit in the neutral community model was higher in surface layers than in subsurface layers of all habitats (Fig. S4C), suggesting that microbial communities were more governed by neutral processes in the surface layer, irrespective of habitat.

**FIG 4:**
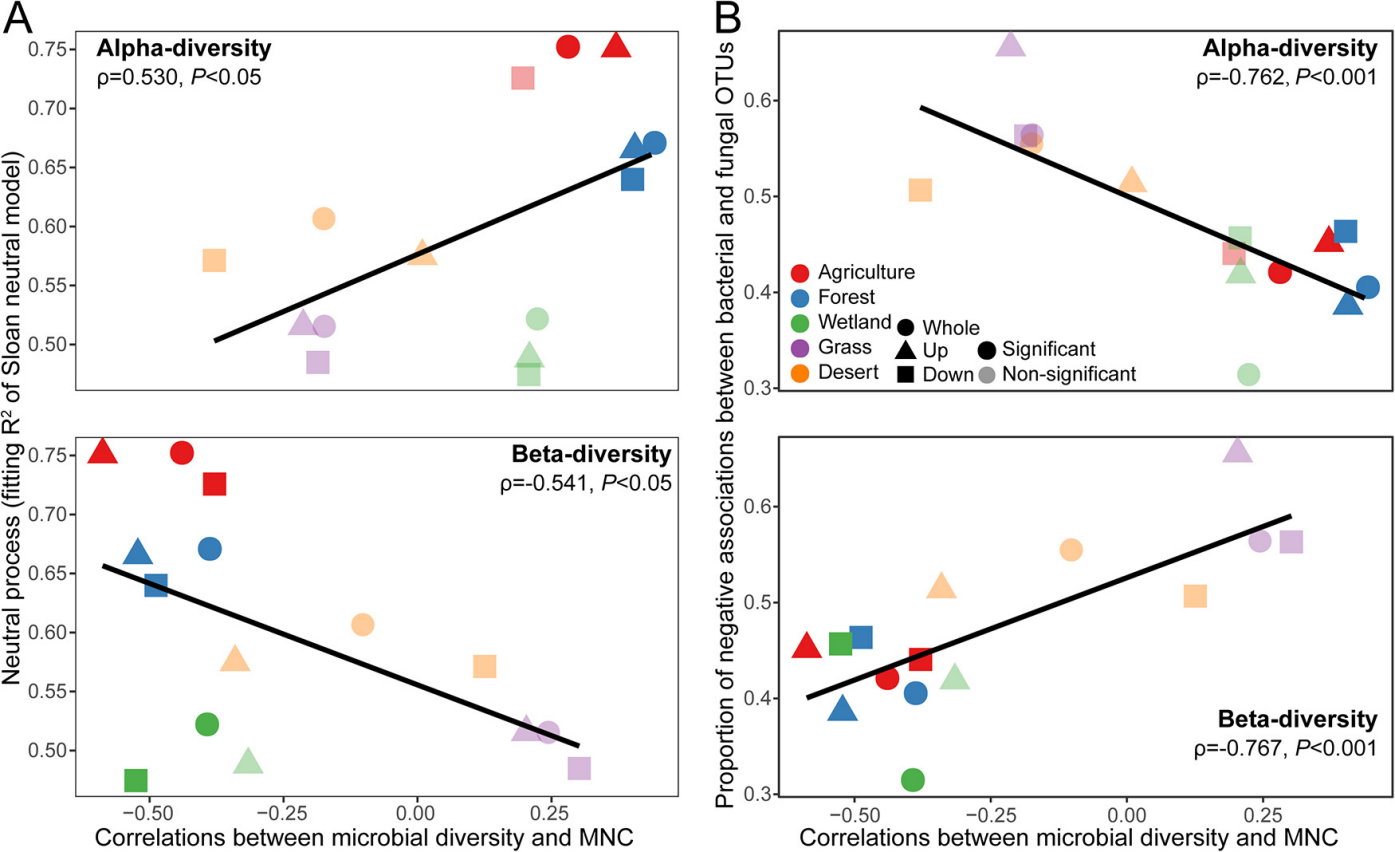
Relationships between the correlation coefficients of soil microbial diversity-MNC relationships and internal community factors. (A) Relationships between the proportions of negative associations between bacterial and fungal OTUs and the correlation coefficients of soil microbial diversity-MNC relationships in different habitats and soil layers, via Pearson’s correlation analysis. (B) Relationships between neutral processes (fitting *R*^2^ in a neutral model) and the correlation coefficients of soil microbial diversity-MNC relationships in different habitats and soil layers via Pearson’s correlation analysis. Whole, whole soil profile; Up, surface soil (0- to 15-cm depth); Down, subsurface soil (15- to 30-cm depth); Significant, significant Pearson’s correlations between microbial diversity and MNC (*P < *0.05); Nonsignificant, nonsignificant Pearson’s correlations between microbial diversity and MNC (*P > *0.05).

We further evaluated how the neutral assembly processes influenced the microbial diversity-MNC relationships. We observed significant relationships between the fit of the neutral model (*R*^2^) and the correlation coefficients of the microbial diversity-MNC relationships (Fig. 4A), indicating that the habitat more governed by neutral processes exhibited a stronger diversity-MNC relationship. Second, we estimated how the balance between positive and negative bacterial-fungal associations correlated with diversity-MNC relationships. Interestingly, the proportions of negative associations between bacterial and fungal taxa were strongly and negatively (or positively) correlated with the correlation coefficients of the microbial diversity-MNC relationships (Fig. 4B and Table 1). In other words, negative bacterial-fungal associations relative to positive ones modified the diversity-function link, whereas there were no significant relationships (*P > *0.1) between the microbial diversity-MNC correlation coefficients and the negative associations among all taxa (Fig. S5A) within bacterial taxa (Fig. S5B) or fungal taxa (Fig. S5C). In addition, no significant correlations (*P > *0.05) were detected between the soil pH-moisture (Fig. S5D and E) and the microbial diversity-MNC relationships.

**TABLE 1:** Associations among all OTUs, within bacterial OTUs and fungal OTUs, and between bacterial and fungal taxa in different habitats and soil layers^*a*^

Habitat	Layer	All			Bacterial			Fungal			Bacterial-fungal		
		Neg	Pos	Pro	Neg	Pos	Pro	Neg	Pos	Pro	Neg	Pos	Pro
Agricultural field	Whole	164,927	259,619	0.388	138,192	212,108	0.394	109	10,822	0.010	26,626	36,689	0.421
Forest	Whole	49,985	94,015	0.347	45,676	82,842	0.355	16	4,860	0.003	4,293	6,313	0.405
Wetland	Whole	34,175	64,190	0.347	32,944	59,761	0.355	3	1,750	0.002	1,228	2,679	0.314
Grass	Whole	130,126	235,602	0.356	116,905	221,191	0.346	26	4,216	0.006	13,195	10,195	0.564
Desert	Whole	17,418	52,058	0.251	16,970	49,556	0.255	3	2,145	0.001	445	357	0.555
Agricultural field	U	15,386	33,637	0.314	13,467	28,226	0.323	0	3,080	0.000	1,919	2,331	0.452
	D	26,347	50,840	0.341	21,427	42,157	0.337	5	2,444	0.002	4,915	6,239	0.441

Forest	U	1,830	4,913	0.271	1,628	4,188	0.280	2	407	0.005	200	318	0.386
	D	1,436	4,568	0.239	1,346	3,778	0.263	1	687	0.001	89	103	0.464
Wetland	U	777	1,699	0.314	709	1,547	0.314	1	59	0.017	67	93	0.419
	D	545	1,288	0.297	508	1,159	0.305	0	85	0.000	37	44	0.457
Grass	U	15,069	42,788	0.260	13,609	40,828	0.250	4	1,195	0.003	1,456	765	0.656
	D	9,541	38,090	0.200	8,507	36,206	0.190	0	1,082	0.000	1,034	802	0.563
Desert	U	1,316	4,846	0.214	1,259	4,510	0.218	1	283	0.004	56	53	0.514
	D	403	1,294	0.237	363	1,059	0.255	1	197	0.005	39	38	0.507

a Values are numbers of associations (Spearman’s correlation) among all OTUs (All), within bacterial OTUs (Bacterial), within fungal OTUs (Fungal), and between bacterial and fungal taxa (Bacterial-fungal) in different habitats and soil layers. Neg, negative association; Pos, positive association; Pro, the proportion of negative associations; Whole, whole soil profile; U, surface soil (up) (0- to 15-cm depth); D, subsurface soil (down) (15- to 30-cm depth).

10.1128/mSystems.01052-20.6 FIG S5 Relationships between the proportions of negative associations among all OTUs (A), within bacterial OTUs (B), within fungal OTUs (C), between soil pHs (D), and between moisture levels (E) and the correlation coefficient of soil microbial diversity-multinutrient cycling (MNC) relationships for *α*-diversity (top) and community composition (β-diversity) (bottom) in different habitats and soil layers. The correlation coefficient and significance of the multidiversity-MNC relationship were estimated using Pearson’s correlation analysis. Whole, whole soil profile; Up, surface soil (0- to 15-cm depth); Down, subsurface soil (15- to 30-cm depth); Significant, significant correlations between multidiversity and MNC (*P < *0.05); Nonsignificant, nonsignificant correlations between multidiversity and MNC (*P > *0.05). Download FIG S5, TIF file, 1.3 MB. Copyright © 2021 Jiao et al. 2021 Jiao et al. https://creativecommons.org/licenses/by/4.0/ This content is distributed under the terms of the Creative Commons Attribution 4.0 International license.

**FIG 5:**
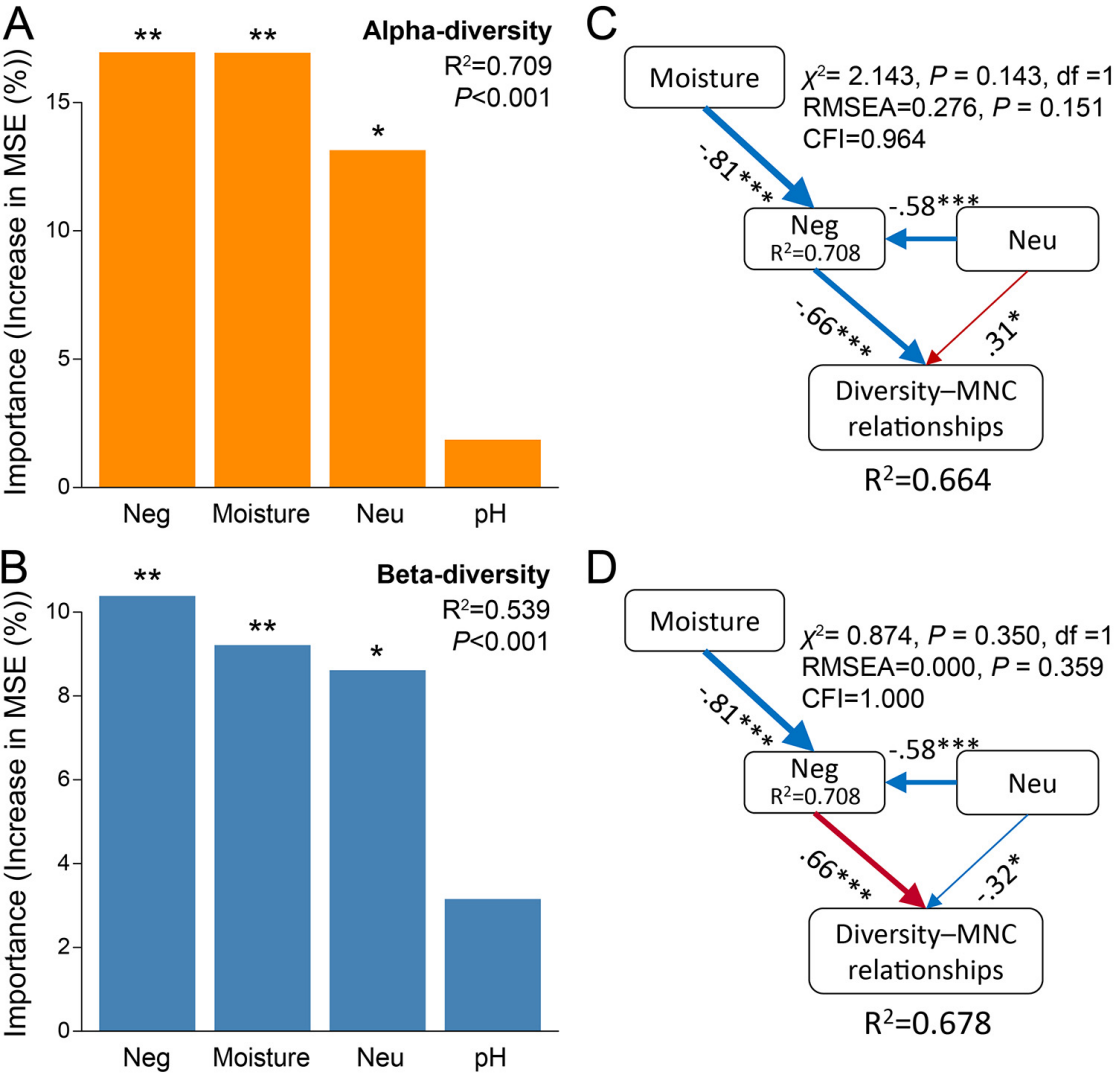
Potential major drivers of the relationship between microbial diversity and the soil MNC. (A, B) Random forest mean predictor importance of negative associations between bacterial and fungal taxa (Neg), neutral community assembly processes (Neu), soil pH, and moisture for the correlation coefficients of the microbial diversity-MNC relationships for *α*-diversity (A) and community composition (β-diversity) (B). The accuracy importance measure was computed for each tree and averaged over the forest (5,000 trees). Percentage increases in the MSE (mean squared error) of variables was used to estimate the importance of these predictors, and higher MSE percentages imply more important predictors. (C, D) Structural equation model describing the effects of different factors on the microbial diversity-MNC relationships for *α*-diversity (C) and community composition (D). Numbers adjacent to arrows are indicative of the effect size of the relationship. *R*^2^ denotes the proportion of variance explained. Red arrows represent positive paths, and blue arrows represent negative paths. Significance levels are as follows: *, *P < *0.05; **, *P < *0.01; ***, *P < *0.001. RMSEA, root mean square error of approximation; CFI, comparative fit index.

We applied a random-forest (RF) analysis to identify the major contributors to the microbial diversity-MNC relationships. We observed that the negative associations between bacterial and fungal taxa made a major contribution to predicting the microbial diversity-MNC relationships (Fig. 5). We then conducted structural equation modeling (SEM) to verify this observation (Fig. S6). The structural equation models had a good fit with the χ^2^ test, the root mean square error of approximation (RMSEA), the comparative fit index (CFI), and their *P* values. Overall, the models explained 66.4 and 67.8% of the variance found in the microbial diversity-MNC relationships for *α*-diversity and community composition, respectively (Fig. 5C and D). In support of our earlier observations, we found the strongest and direct negative correlations between negative bacterial-fungal associations and the microbial diversity-MNC relationships. In addition, there was a negative relationship between the neutral processes and negative bacterial-fungal associations.

10.1128/mSystems.01052-20.7 FIG S6 *A priori* structural-equation model describing the effects of different factors on the relationships between microbial diversity and soil multinutrient cycling. Neg, negative associations between bacterial and fungal taxa; Neu, neutral community assembly processes. Since our sampling sites for each habitat were evenly distributed along the transect of the Hexi Corridor to control the effect of spatial scale, the spatial and climatic variables were not included in the model. Download FIG S6, TIF file, 0.2 MB. Copyright © 2021 Jiao et al. 2021 Jiao et al. https://creativecommons.org/licenses/by/4.0/ This content is distributed under the terms of the Creative Commons Attribution 4.0 International license.

## DISCUSSION

Soil microbial diversity promotes multifunctionality in natural terrestrial ecosystems (33, 34) but may be context dependent across complex ecosystems, with community assembly processes and balance between positive and negative interaction potentially controlling this context dependency. Therefore, characterizing determining factors is crucial for advancing the prediction and regulation of biodiversity-ecosystem function (BEF) relationships. Here, we reveal that community assembly processes and the balance between positive and negative bacterial-fungal associations were clearly linked with the microbial diversity-MNC relationships. The habitats less governed by neutral processes and dominated by negative bacterial-fungal associations exhibited stronger diversity-MNC relationships.

High soil microbial diversity can promote ecosystem multifunctionality and regulate multifunctionality resistance to climate change and fertilization in natural terrestrial ecosystems (33, 35, 36). Growing evidence for a strong link between soil biodiversity and multiple ecosystem functions has been reported (33, 34, 36–38); however, these relationships have not yet been compared across habitats. Here, we observed that the microbial *α*-diversity and MNCs in desert soils were lower than in other habitats along the Hexi Corridor. This is supported by a previous study demonstrating that microbial communities in desert soils had the lowest levels of taxonomic diversity and gene abundances associated with nitrogen, potassium, and sulfur metabolism, compared to those of forests, grasslands, and tundra habitats (39). Moreover, the significant differences in the microbial community compositions among the five habitats and between the two soil layers were in agreement with recent studies that demonstrated strong habitat-specific patterns of microbial β-diversity in soil ecosystems (40, 41). Specifically, we did not observe significant microbial diversity-MNC relationships in desert and grassland soils. Since oases were scattered along the narrow desert belt in arid regions, most of the grassland soil samples were collected from desert grassland in this study (40). In this case, the nonsignificant microbial diversity-MNC relationships in desert and grassland soils imply that desertification or grassland degradation might weaken the diversity-function relationship.

Positive effects of local-scale biodiversity on ecosystem functions have been demonstrated in theoretical, experimental, and observational studies across different types of ecosystems and habitats (21, 24, 34, 36, 38, 42). Recently, the viewpoint that β-diversity is important in the context of multifunctionality was proposed (1). In the present study, we observed that community composition showed higher correlation coefficients with MNC than with *α*-diversity, suggesting the major role of microbial community composition on soil nutrient cycling. This supports the notion that community composition might be more important than community richness (43, 44). In addition, the role of microbial community composition in regulating ecosystem functions is related to variations in local-scale diversity, which may scale up to large-scale changes in the provisioning of multiple ecosystem functions (45, 46). Multifunctionality resistance to climate change and nitrogen fertilization is regulated by soil bacterial and fungal community compositions in natural ecosystems (47). Bacterial and archaeal β-diversities were strongly related to soil multinutrient cycling in a 30-year chronosequence of a reforestation ecosystem (48). S. Jiao et al. (41) demonstrated that soil multinutrient cycling in waterlogged rice fields was associated with bacterial β-diversity, potentially attributable to the metabolic cooperation via syntrophy between bacterial groups under oxygen-limited conditions (41). Based on these cases, our results therefore support the perspective that community composition is closely linked to the provisioning of multiple ecosystem functions (multinutrient cycling, for example) (1). This may improve our understanding of BEF relationships under various natural and anthropogenic influences.

Biodiversity is a central topic in ecology, because the dramatic loss in biodiversity may reduce ecosystem functions and services (49). It is crucial to reveal the factors used to determine the strength of microbial diversity-function relationships in complex terrestrial ecosystems, considering the intrinsic linkages between assembly processes and species interactions. Understanding the assembly mechanisms of belowground microbial communities is crucial for understanding the maintenance and generation of terrestrial microbial diversity (50–52). Previous studies showed that microbial biogeography exhibited strong habitat-specific patterns (40, 41). In the present study, we found that the assembly of microbial communities in agricultural soils was more governed by neutral (e.g., stochastic) processes than that in other natural habitats, indicating that long-term cultivation and human-managed activities (53) might enhance the stochastic influx and dispersal of microorganisms. Our result supported prior studies reporting that stochastic processes were stronger in crop fields than in grassland (54). Additionally, we observed that neutral processes were stronger in surface soils than in subsurface layers, irrespective of habitat. This might be due to the difference in dispersal between surface and subsurface soils (55). Particularly, available nutrient substrates usually decrease with soil depth because of the reduced input of plant litter and root exudates (56). High available nutrients introduce more resources to soil microbes, which can improve the ability of microorganisms to disperse and in turn increase the dominance of stochastic (e.g., neutral) processes (3). Therefore, our results suggest that soil depth may affect the microbial community assembly processes.

Species interactions are considered to be deterministic (niche-based) assembly processes governing community structure (6, 57, 58). In the present study, the SEM results showed a negative relationship between the neutral processes and negative bacterial-fungal associations, indicating that negative relationships tended to be lower when communities were driven primarily by neutral processes. That is, positive bacterial-fungal associations were more widespread under stronger stochastic processes. This may be supported by a previous study demonstrating that soil microbial cooccurrence associations are higher when communities are driven primarily by stochastic processes (e.g., dispersal limitation) (59). Stochastic processes involve random birth, death, and dispersal events; therefore, species tend to cooccur when random changes and increased influxes of species are not associated with environmentally derived fitness (6, 7). Previous studies have demonstrated that community assembly processes were associated with the microbial interactions (e.g., cooccurrence associations based on the correlation network analysis) in soil (59) and river (60) systems. In addition, a global soil microbiome study provided evidence for strong bacterial-fungal antagonism, suggesting the role of species interactions in shaping microbial communities (12). On this basis, our findings more specifically uncover the intrinsic linkages between assembly processes and microbial associations (e.g., positive or negative) and suggest that the balance between positive and negative bacterial-fungal associations is strongly related to community assembly processes.

Furthermore, we observed that the neutral processes substantially influenced the magnitude and direction of the diversity-MNC relationships. The habitats more governed by neutral processes exhibited stronger positive or negative diversity-MNC relationships for *α*-diversity or community composition, respectively. This may be explained by two mechanisms: (i) immigration can add species or change species composition in a way that increases the biodiversity effect on functions through the sampling effect if immigration from the regional pool brings new species carrying traits that affect ecosystem functioning and that were not present in the initial community (10) and (ii) a higher relative balance of a deterministic process (and thus the lower importance of neutral ones) can decrease the biodiversity effect on ecosystem function by a dilution effect if species selected by deterministic processes are not the ones affecting ecosystem functioning (10). Thus, a lower influence of deterministic processes (and thus a higher influence of stochastic ones) might reduce this dilution effect and strengthen the BEF relationship, as observed in the present study. A previous study used a model to assess how the relative balance between stochastic and deterministic processes affect a generic biogeochemical function and revealed that higher dispersal led to decreases in biogeochemical function due to the increased abundance of poorly adapted organisms (61). The inconsistency might be attributed to the distinct models, and we considered only the effect of microbial diversity on biogeochemical functions. Uniquely, our study builds the linkage between the mechanisms underlying community assembly and the BEF relationships and suggests that neutral processes governing the distinct assembly patterns of a cross-habitat microbial community may enhance the functional contributions of ecological communities. This highlights the potential roles of community assembly mechanisms in generating and sustaining the multiple-nutrient cycling of terrestrial ecosystems.

Species interactions play important roles in stimulating ecosystem processes (62). In the present study, we observed that the proportions of negative associations between bacterial and fungal taxa were clearly linked with the microbial diversity-MNC relationship when we considered environmental factors and community assembly processes. It indicated that the balance between positive and negative bacterial-fungal associations underpinned the context dependency of the microbial diversity-MNC relationship. Negative species associations may be due to antagonistic biological interactions (63), including competition. It is thus conceivable that a high number of antagonistic or competitive interactions between bacterial and fungal taxa led to the weak microbial diversity-MNC relationships observed in grassland and desert habitats, which was supported by the SEM results. Competition was a major type of interaction between fungi and bacteria in soil (19, 20, 64, 65). For example, some soil-derived fungal and bacterial species may synthesize antibiotics and showed antagonistic effects (26, 27) which had consequences for microbial community assembly (12). A study has shown that strongly hierarchical competitive network communities comprising strong competitors exhibit a negative diversity-function relationship (19). Positively interacting species can increase functional community performance due to niche partitioning by distinct resources (15, 16). Soil organisms with similar environmental preferences may form strongly connected ecological clusters in ecological networks, with major implications for ecosystem functioning (66, 67). Previous studies showed that fungal-bacterial interkingdom associations may enhance ecosystem functioning related to nutrient cycling in grasslands (31) and promote plant health in the model plant *Arabidopsis* (68). Thus, these studies may support our conclusion that the balance between positive and negative associations were connected to the link between soil microbial diversity and multinutrient cycling.

One potential limitation of this study is that the negative/positive associations between taxa should not be interpreted as a proof of competition/facilitation, which was based only on correlation (69). Correlation analyses are only a simplistic representation of a complex system, although they are frequently used to investigate microbial interconnection patterns (70–72). In addition, species associations that are based on correlations can yield spurious results and cannot be automatically interpreted as interactions (63). Consequently, it may not be possible to comprehensively depict the microbial interactions under real-world conditions. However, the information about negative/positive correlations between taxa is still essential for estimating potential species interrelationships within complex environments and, in turn, for revealing the influence of microbial interconnection complexity on biodiversity-driven ecosystem functioning (67).

### Conclusions.

Our study highlights that the balance between positive and negative bacterial-fungal associations is clearly linked with the strength of the relationships between soil microbial diversity and multiple nutrients cycling across different habitats. Changes in both the level of neutrality and the proportion of negative bacterial-fungal associations are linked to the magnitude and the direction of the diversity-MNC relationship. These findings reveal the potential factors underpinning the context dependency of the microbial diversity-MNC relationship, which makes it possible to predict the ecological consequences for the biodiversity-function relationship in the future by uncovering how soil microbial assembly and associations are likely to respond to the climate and land use changes. For example, the nonsignificant microbial diversity-MNC relationship in desert soils suggests that desertification might weaken the microbial diversity-function relationship, along with increasing negative bacterial-fungal associations. The strongest neutral process of microbial assembly in agricultural soils implies that the conversion from natural habitats to agricultural fields (e.g., the conversion of grassland for agricultural crops, the conversion of forests for crop and wetland degradation) might enhance the stochastic influx and dispersal of microorganisms and strengthen the biodiversity-MNC relationship. Overall, our results represent a considerable advancement in facilitating the management of the functioning of microbial communities for improving human well-being, in addition to informing strategies based on community assembly and microbial interactions.

## MATERIALS AND METHODS

### Site description.

The study site extended from 36°56′N to 40°34′N and from 94°37′E to 103°31′E along the Hexi Corridor in the northwestern portion of Gansu Province and to the west of the Yellow River in China (see Fig. S1 in the supplemental material). The Hexi Corridor is a long belt between the South Mountains (including Mt. Qilian and Mt. Aerjin) and the North Mountains (including Mt. Mazong, Mt. Heli, and Mt. Longshou). Due to the distribution of oases scattered along the narrow desert belt, the Hexi corridor contains a variety of soil ecosystems (32).

Here, we selected five habitats: an agricultural field, forest, wetland, grassland, and desert, according to a vegetation map at a scale of 1:1,000,000 (Data Centre for Resources and Environmental Sciences, Chinese Academy of Sciences [RESDC], http://www.resdc.cn). The dominant species in these habitats included *Zea mays* (agricultural field), *Calligonum* spp., *Stipa* spp., *Leymus* spp., and *Achnatherum* spp. (wetland, grassland, and desert), and *Populus* spp. (forest). The dominant soil types were aripsamment and calciorthids, which have a loose structure and low organic matter content. The climate of this region is predominantly semiarid.

### Sample collection.

Field sampling was conducted during July and August 2017 near the period of the highest aboveground plant biomass. To ensure appropriate spatial scale, the sampling sites for each habitat were evenly distributed along the transect of the corresponding region. This meant that some sampling sites for different habitats were spaced less than 5 km apart; hence, some of these sites overlapped on the map (Fig. S1). In total, 126 sites were selected; 37 were from an agricultural field, 28 were from a forest, 15 were from a wetland, 26 were from a grassland, and 20 were from a desert.

At each site, three plots were sampled; each plot had an area of 100 m^2^. Five soil cores (2.5-cm diameter) were combined per plot and were taken at depths of 0 to 15 cm and 15 to 30 cm (56, 73). The soil cores from the three plots for a given soil depth layer were then mixed thoroughly to generate the final soil samples. All soil samples were delivered to the laboratory in sterile plastic bags on dry ice and were sieved through a 2.0-mm mesh to remove plant debris and rocks. A portion of each soil sample was stored at 4°C for the analysis of nutrient factors. Aliquots of soil samples were stored at −20°C for subsequent DNA extraction. One subsurface desert sample was abandoned due to DNA extraction failure. Therefore, a total of 251 soil samples at 126 sites with two soil depth layers were used for this study. Standard testing methods were applied to measure soil pH, moisture, soil organic carbon, dissolved organic carbon, microbial biomass carbon, nitrate-nitrogen, ammonium-nitrogen, microbial biomass nitrogen, available phosphorus, and available potassium, as previously described (50, 71, 74). Detailed descriptions are provided in the supplemental material. We acknowledge that many important environmental parameters (e.g., the types of carbon/nitrogen/phosphorus, the hydrology, temperature, oxygen stress, microsite structure, anion/cation/micronutrients, etc.) were not measured in this study, and these should be considered in future work.

### Microbial DNA processing.

Bacterial and fungal diversity scores were obtained via high-throughput sequencing of the PCR amplicons of the 16S rRNA and internal transcribed spacer (ITS) genes. Briefly, the total genomic DNA was extracted from the soil samples (0.5 g) using a FastDNA SPIN kit for soil (MP Biochemicals, Solon, OH, USA). The microbial communities were profiled by targeting the V4-V5 region of the 16S rRNA gene for bacteria and the ITS1 region of the 18S rRNA gene for fungi. The target sequences were amplified by PCR using the primer pairs 515F/907R (bacteria) and ITS5-1737F/ITS2-2043R (fungi) (75, 76). Sequencing was conducted on an Illumina HiSeq2500 platform (Illumina Inc., San Diego, CA, USA).

The acquired sequences were filtered for quality according to the method of J. G. Caporaso et al. (77). Sequences were assigned to their corresponding samples according to the barcode and then quality trimmed with a threshold of average Phred quality scores of higher than 20. Any chimeric sequences were removed with the USEARCH tool based on the UCHIME algorithm (78). The sequences were split into groups according to their identity and assigned to operational taxonomic units (OTUs) at a 3% dissimilarity level using the UPARSE pipeline (78). The OTUs with no more than two sequences were removed, and their representative sequences were classified within the SILVA database (release 128) for bacteria (79) and UNITE plus INSD (UNITE and the International Nucleotide Sequence Databases; release 7) for fungi (80). Counts of individual OTUs were scaled by the total number of reads in each sample to account for sequencing biases using the R package DESeq2 (81). This measure of normalized abundance allows samples with various read counts to be compared (82) and is widely applied to high-throughput sequencing data (82, 83).

### Soil microbial diversity analysis.

To obtain a multidiversity index, we combined soil microbial diversity characteristics by averaging the standardized scores of bacterial Shannon diversity and fungal richness. The scores standardized to a common scale ranging from 0 to 1 were calculated according to the following formula: STD = (*X* − *X*_min_)/(*X*_max_ − *X*_min_), where STD is the standardized variable and *X*, *X*_min_, and *X*_max_ are the target variable and its minimum and maximum values across all samples, respectively. This multidiversity index is largely used and accepted in the current biodiversity function literature (34, 37, 84). The microbial β-diversity was quantified using the first axis of a nonmetric multidimensional scaling (NMDS) analysis of Bray-Curtis dissimilarities by combining the bacterial and fungal communities (85). Here, the analysis was based on combining the bacterial and fungal communities, including all of the bacterial and fungal OTUs. Before the combination, the bacterial and fungal communities were standardized to a total abundance of 1.

### Evaluation of soil ecosystem function.

Ecosystems perform multiple simultaneous functions and services, rather than a single measurable process. Given that nutrient cycling is the most important soil ecosystem process for supporting human welfare (62, 86), we estimated the multinutrient cycling index (MNC) to evaluate soil ecosystem functions (41, 48). This index comprised information for 8 soil nutrient variables in relation to carbon (soil organic carbon, dissolved organic carbon, and microbial biomass carbon), nitrogen (nitrate-nitrogen, ammonium-nitrogen, and microbial biomass nitrogen), phosphorus (available phosphorus), and potassium (available potassium) cycling. These variables constitute an integrated proxy for nutrient cycling and are important determinants of ecosystem functioning in terrestrial ecosystems (33, 34, 84). For example, nitrogen and phosphorus are the nutrients that most frequently limit primary production in terrestrial ecosystems (87). In addition, potassium is the third essential macronutrient required by plants; it participates in a range of biological activities, such as protein synthesis, enzyme activation, and photosynthesis, that maintain or improve plant growth (88). We acknowledge that some important functions, such as soil process rates, are inevitably unmeasured, and future studies are encouraged to include more essential functions for comprehensive understanding of ecosystem functioning. To derive a quantitative MNC value for each site, we averaged the standardized scores (a common scale ranging from 0 to 1) of all individual nutrient variables. This method was used to quantify soil multinutrient cycling because it is a straightforward and interpretable measure of a community’s ability to sustain multiple functions simultaneously (33, 34, 84).

### Ecological analysis.

The NMDS analysis was performed to visualize the sample relationships across different habitats. An analysis of similarities (ANOSIM) was used to determine significant differences in microbial community composition across different habitats, performed using the anosim function in the vegan package in R (89). Multiple-comparison *post hoc* tests for Kruskal-Wallis analysis were used to test for significant differences in microbial diversity and soil multinutrient cycling among different habitats, performed using the kruskal function in the agricolae package in R (90). Pearson’s correlation analysis was used to estimate the relationship between microbial diversity and multinutrient cycling, performed using the cor.test function in the stats package in R (91).

### Neutral modeling.

A Sloan neutral community model was used to determine the contribution of neutral processes to microbial community assembly (92). The model predicts that abundant taxa are more likely to be dispersed by chance and widespread across a metacommunity, while rare taxa are lost in different local communities due to ecological drift. The neutral model is fit to the relationship between the frequency with which taxa occur in a set of local communities and their abundance across the wider metacommunity by estimating the parameter describing the migration rate (*m*), a measure of dispersal limitation. Higher *m* values indicate that microbial communities are less dispersal limited (92, 93). The formula (59) is Freq=1 − I[1/N,N×m×p,N×m(1 − p)], where Freq is taxon occurrence frequency, *N* is the number of individuals per community, *p* is the taxon relative abundance, and *I*[] is the probability density function of the beta distribution. *R*^2^ indicates the fit of the parameter based on nonlinear least-squares fitting. The overall fit of the model to the observed data was assessed by comparing the sums of squares of residuals, SS_err_, with the total sum of squares, SS_total_: model fit = 1 − SS_err_/SS_total_ (generalized *R*-squared) (93). Higher *R*^2^ values indicate a higher contribution of neutral processes to microbial community assembly. In the present study, we used the fit of the neutral model (*R*^2^) to infer the neutral assembly processes. One point should be noticed, i.e., that stochastic processes do not exactly incorporate a neutral process, although a few recent researchers have applied neutral-theory-based process models to infer the stochastic processes (57, 94). Here, the analysis was based on combining the bacterial and fungal communities, which were, respectively, standardized to a total abundance of 1.

### Microbial association analysis.

To explore the potential interactions among species, we estimated the associations among all the members of the bacterial and fungal communities in different habitats and soil layers. Robust correlations were estimated via Spearman’s correlation analysis with false-discovery rate (FDR)-corrected *P* values of <0.01, which were used to reflect the negative (Spearman’s correlation coefficient [*ρ*] < 0) or positive (*ρ* > 0) associations among microbial taxa. The proportion of negative associations meant negative associations divided by the total associations. To avoid random effects of rare taxa, only taxa detected in more than 60% of the soil samples in each habitat (e.g., different habitats and layers) were used for the correlation analysis (95). To test whether the outcomes were sensitive to the choice, we also estimated the microbial associations based on the 50% threshold. Similar results with the 60% threshold were observed (data not shown), indicating that the outcomes were not sensitive to the choice of threshold.

### RF modeling.

We first evaluated Pearson’s correlations between the strength of microbial diversity-MNC relationships (correlation coefficients) and (i) the proportions of negative associations between bacterial and fungal taxa, within all taxa, within bacterial taxa, and within fungal taxa; (ii) the neutral community assembly processes (*R*^2^ of the neutral model); and (iii) environmental variables, including soil pH and moisture. Additionally, random-forest (RF) analysis was performed to identify the main factors influencing the microbial diversity-MNC relationships (33, 96). In the RF models, negative associations between bacterial and fungal taxa, neutral community assembly processes, soil pHs, and moisture levels served as predictors for the correlation coefficients of the microbial diversity-MNC relationships. To estimate the importance of these variables, we used percentage increases in the mean squared error (MSE) of variables: higher MSE percentages imply more important variables (97). The significance of the model was assessed with 5,000 permutations of the response variable by using the A3 package (98). Similarly, the significance of each predictor on the response variables was assessed with 5,000 trees by using the rfPermute package (99).

### SEM.

We then used structural equation modeling (SEM) to evaluate the direct and indirect effects of different factors on the strengths of microbial diversity-MNC relationships. The first step in SEM requires establishing an *a priori* model based on the known effects and relationships among the drivers (Fig. S6). The negative associations between bacterial and fungal taxa, neutral community assembly processes, soil pH, and moisture were considered in the model. Since our sampling sites for each habitat were evenly distributed along the transect of the Hexi Corridor to control the effect of spatial scale, the spatial and climatic variables were not included in the model. We fitted the full model containing all potential paths of our *a priori* model (Fig. S6) and then simplified the model by removing the variable (e.g., soil pH) without any significant relationship (44). Each path removal was accepted if the model quality-based Akaike information criterion (AIC) was improved. The goodness of fit of structural equation models was checked using the following: the χ^2^ test, the root mean square error of approximation (RMSEA), and the comparative fit index (CFI). The model has a good fit when the CFI value is close to 1, RMSEA values are closer to 0, and χ^2^ and RMSEA *P* values are high (traditionally >0.05) (100). With a good model fit, we were free to interpret the path coefficients of the model and their associated *P* values. A path coefficient is analogous to the partial correlation coefficient and describes the strength and sign of the relationship between two variables. SEM was conducted with the lavaan package (101).

All statistical analyses were performed in the R environment (v3.5.1; http://www.r-project.org/), using vegan (89), stats (91), A3 (98), rfPermute (99), lavaan (101), fdrtool (102), Hmisc (103), ggplot2 (104), relaimpo (105), and gplots (106) packages.

### Data accessibility.

The raw sequence data reported in this paper have been deposited in the Genome Sequence Archive (107) and in the Beijing Institute of Genomics (BIG) Data Center (108), BIG, Chinese Academy of Sciences, under BioProject accession no. PRJCA004036 and are publicly accessible at http://bigd.big.ac.cn/gsa.

10.1128/mSystems.01052-20.1 TEXT S1 Supplemental materials and methods. Standard testing methods were applied to measure soil pH, moisture, soil organic carbon, dissolved organic carbon, microbial biomass carbon, nitrate-nitrogen, ammonium-nitrogen, microbial biomass nitrogen, available phosphorus, and available potassium, as previously described. Detailed descriptions of the standard testing methods of soil variables. Download Text S1, DOCX file, 0.04 MB. Copyright © 2021 Jiao et al. 2021 Jiao et al. https://creativecommons.org/licenses/by/4.0/ This content is distributed under the terms of the Creative Commons Attribution 4.0 International license.

10.1128/mSystems.01052-20.9 DATA SET S1 Soil sample IDs, nutrient variables, and locations. Download Data Set S1, XLSX file, 0.05 MB. Copyright © 2021 Jiao et al. 2021 Jiao et al. https://creativecommons.org/licenses/by/4.0/ This content is distributed under the terms of the Creative Commons Attribution 4.0 International license.
