# Supplementary figures and images for: Linking Bacterial-Fungal Relationships to Microbial Diversity and Soil Nutrient Cycling
Source: mSystems. 2021 Mar 23;6(2):e01052-20. doi: 10.1128/mSystems.01052-20 (PMC8546990; doi:10.1128/mSystems.01052-20)

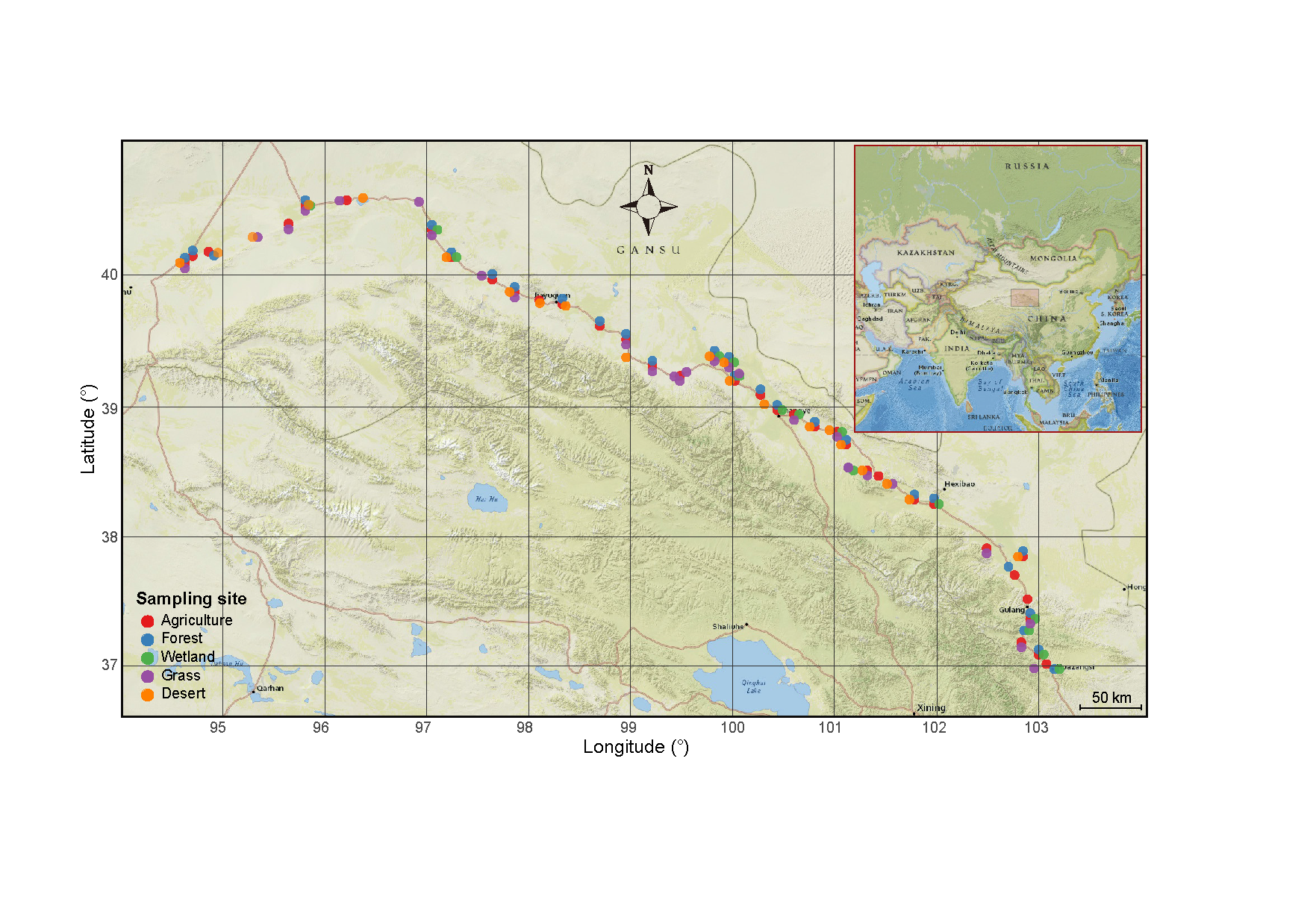

Supplement: FIG S1 [file msystems.01052-20-sf001.tif]

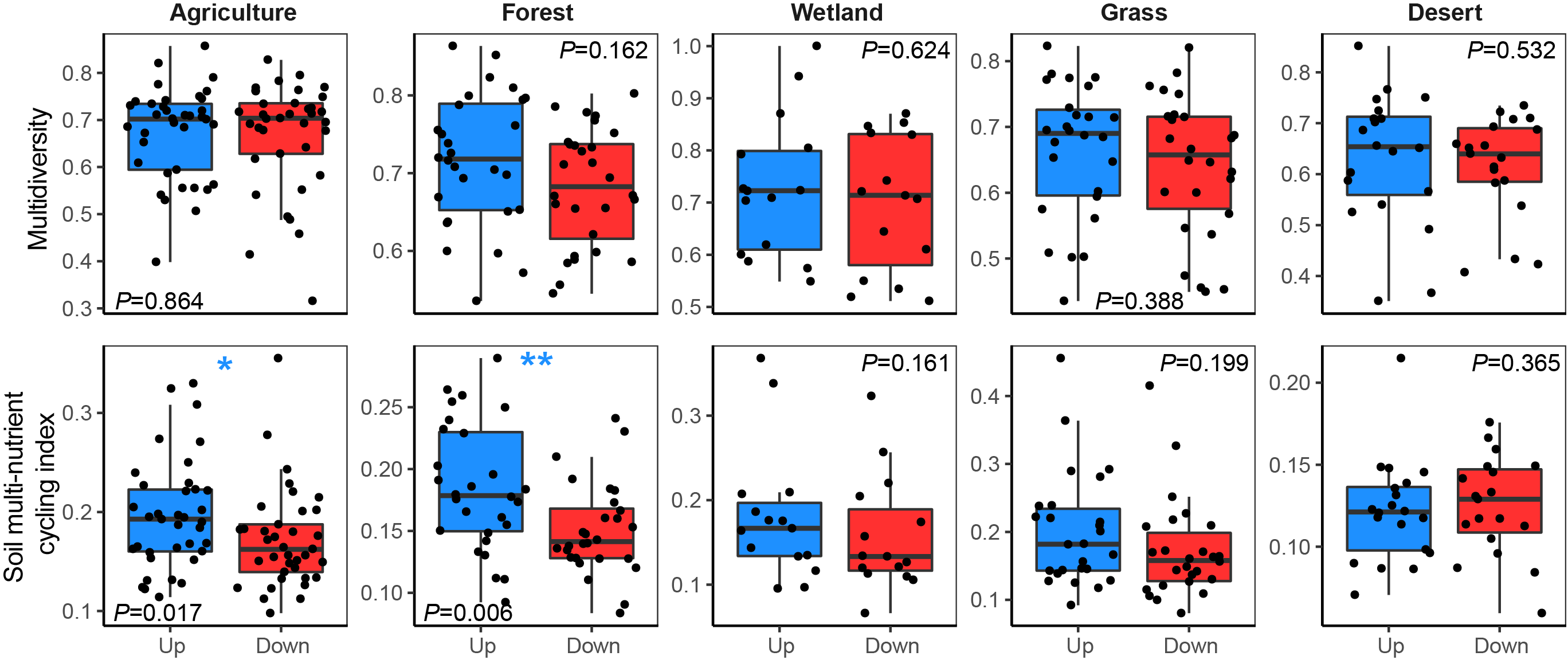

Supplement: FIG S2 [file msystems.01052-20-sf002.tif]

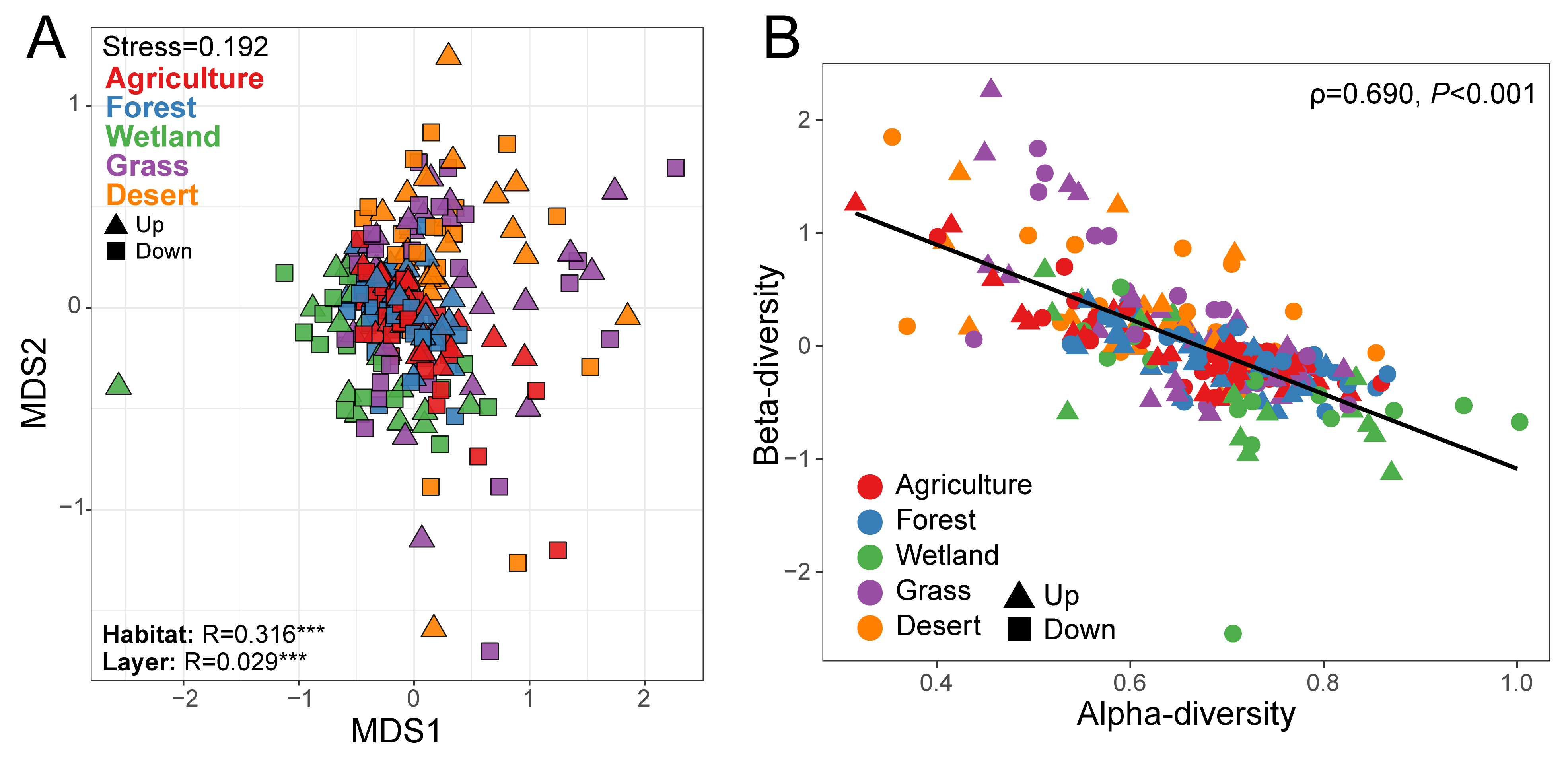

Supplement: FIG S3 [file msystems.01052-20-sf003.tif]

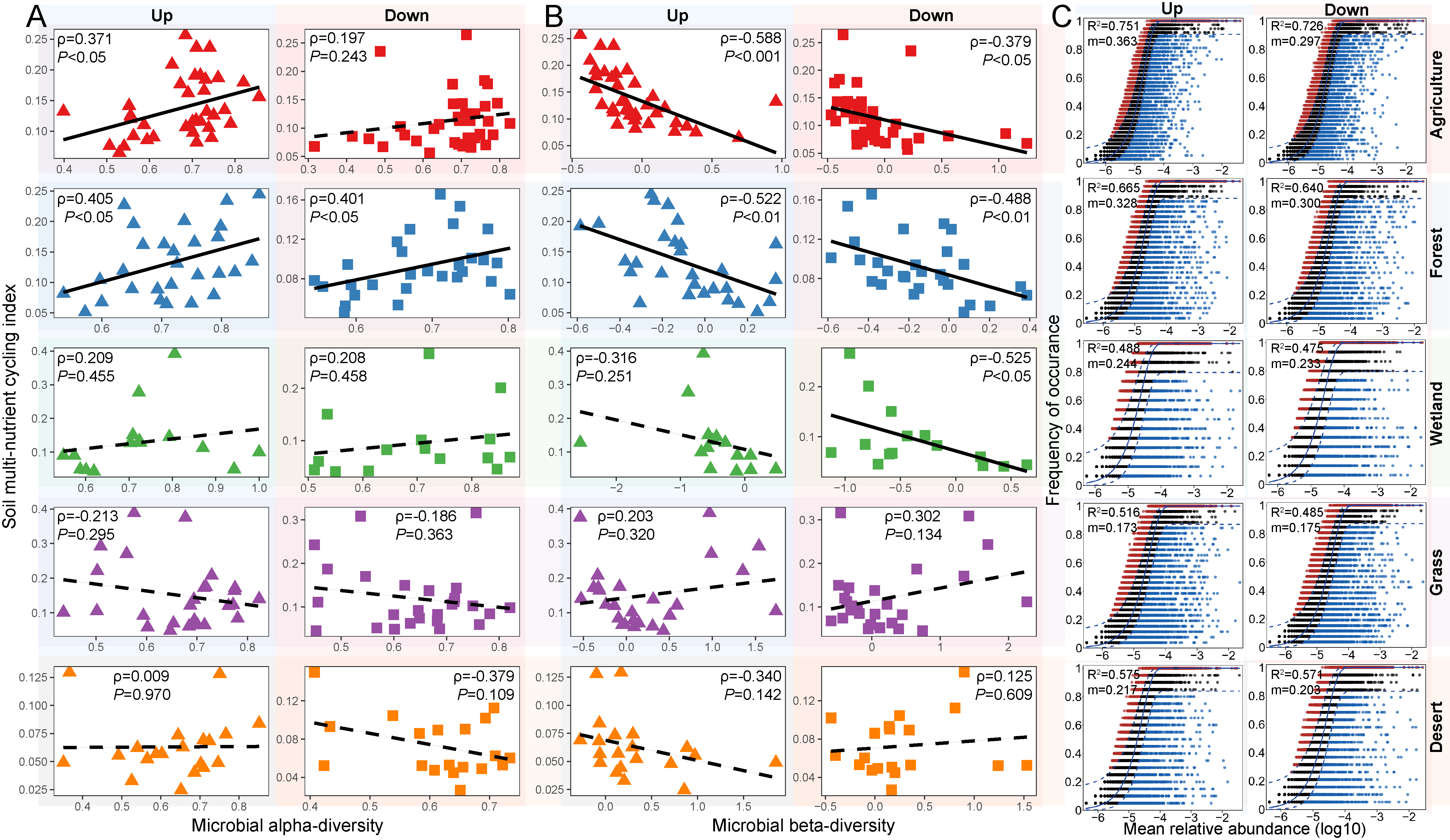

Supplement: FIG S4 [file msystems.01052-20-sf004.tif]

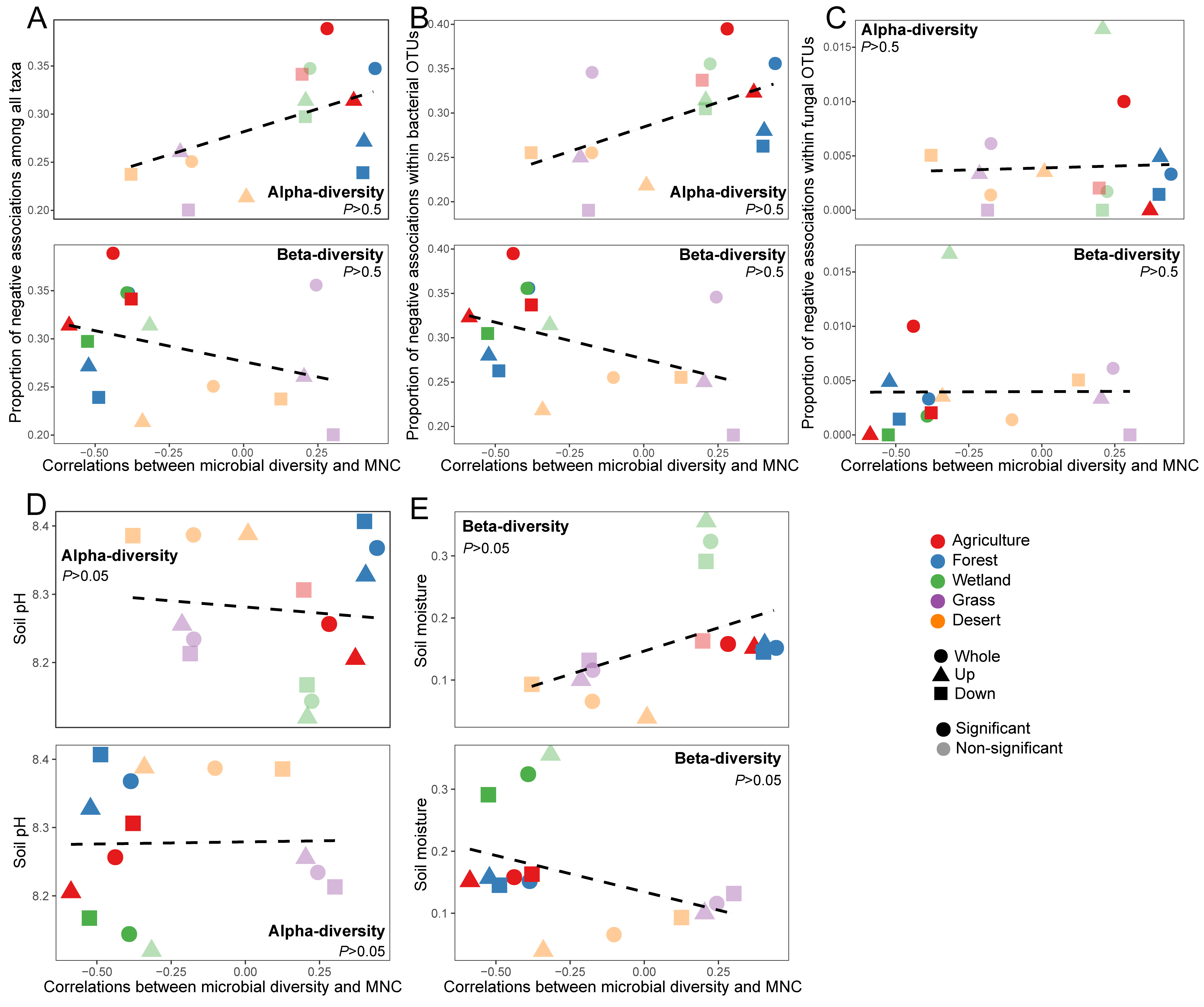

Supplement: FIG S5 [file msystems.01052-20-sf005.tif]

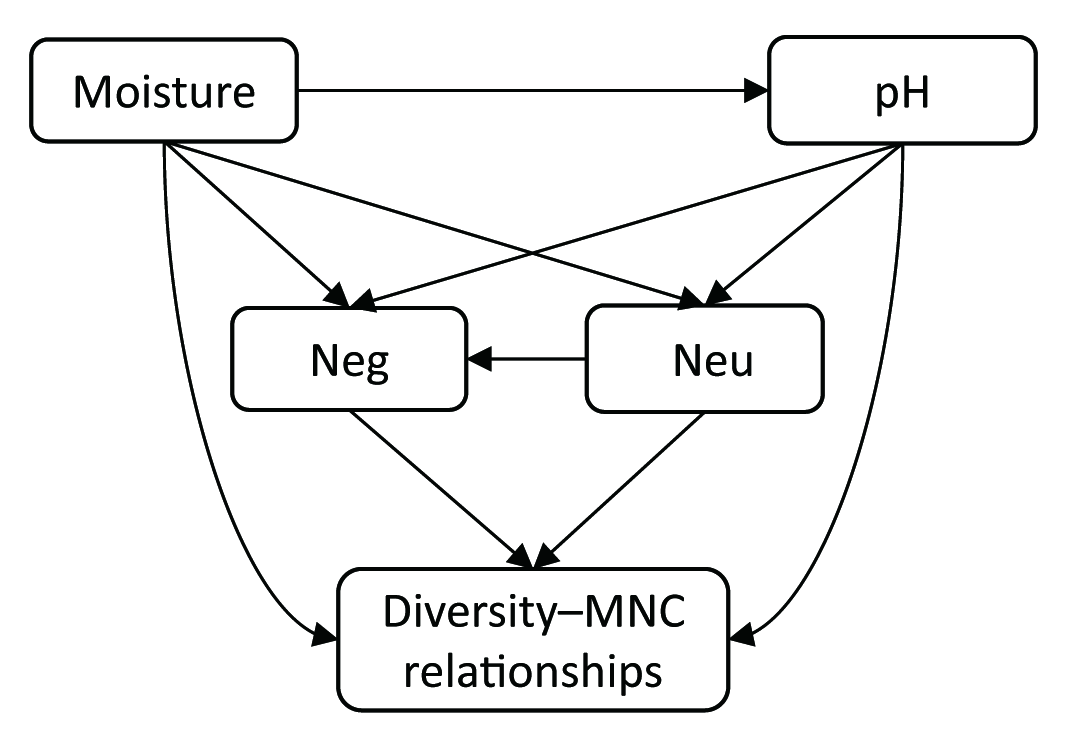

Supplement: FIG S6 [file msystems.01052-20-sf006.tif]
