# Supplementary material for: Linking Bacterial-Fungal Relationships to Microbial Diversity and Soil Nutrient Cycling
Source: mSystems. 2021 Mar 23;6(2):e01052-20. doi: 10.1128/mSystems.01052-20 (PMC8546990; doi:10.1128/mSystems.01052-20)
Supplement: TABLE S1 [file msystems.01052-20-st001.docx]

**Table S1** Variation partitioning analysis of the relative contributions (%) of microbial *α*-diversity and *β*-diversity to variation in multi-nutrient cycling in the different habitats. “Common”, the simultaneous effects of *α*-diversity and *β*-diversity.

|  | *α*-diversity | *β*-diversity | Common |
| --- | --- | --- | --- |
| *Agriculture* | 6.03 | 18.25 | 9.24 |
| *Forest* | 11.13 | 13.51 | 0.21 |
| *Wetland* | 2.51 | 13.63 | 4.05 |
| *Grass* | 1.55 | 4.57 | 5.12 |
| *Desert* | 5.73 | 3.67 | 0.01 |
