# Supplementary material for: Linking Bacterial-Fungal Relationships to Microbial Diversity and Soil Nutrient Cycling
Source: mSystems. 2021 Mar 23;6(2):e01052-20. doi: 10.1128/mSystems.01052-20 (PMC8546990; doi:10.1128/mSystems.01052-20)
Supplement: TEXT S1 [file msystems.01052-20-s0001.docx]

**MATERIALS AND METHODS**

Standard testing methods were applied to measure soil pH, moisture, soil organic carbon, dissolved organic carbon, microbial biomass carbon, nitrate-nitrogen, ammonium-nitrogen, microbial biomass nitrogen, available phosphorus, and available potassium. Soil pH was measured with a pH meter in a 1:2.5 (mass:volume) soil–water suspension. Organic carbon was determined according to potassium dichromate oxidation titration. Dissolved organic carbon was extracted by adding 50 ml of 0.5 M K_2_SO_4_ to 10 g fresh soil, shaking for 1 h, and vacuum filtering through a G4 glass fiber filter with a pore space of 1.2 μm (Fisher), and was determined using a total organic carbon analyzer (Shimadzu, Kyoto, Japan). Ammonium (NH_4_^+^) and nitrate (NO_3_^-^) nitrogen was extracted by K_2_SO_4_ solution, and was determined colorimetrically by automated segmented flow analysis (AAIII; Bran and Luebbe, Germany) using the cadmium column/sulfanilamide reduction method. Available potassium was determined in 1 M ammonium acetate extracts by flame photometry (FP640, INASA, China). Available phosphorus was extracted by 0.5 M NaHCO_3_ and determined using the molybdenum blue method.

Microbial biomass carbon (MBC) and nitrogen (MBN) was measured using the fumigation-extraction method (1, 2). Briefly, for each soil, three of six fresh subsamples (10 g dry weight equivalent) were fumigated with free-ethanol chloroform for 24 h in vacuum desiccators, another three were not fumigated as the control. The soil samples were extracted with 0.5 M K_2_SO_4_ (the ratio of soil/extractant was 1:4) for 30 minutes (300 rpm) in an oscillator. The unfumigated soil samples were also subjected to a similar extraction. The resulting extracts were filtered. MBC in filtrates was determined by potassium dichromate method (1), while MBN was estimated by Kjeldahl method (2).

**Reference:**

1. **Vance ED, Brookes PC, Jenkinson DS.** 1987. An extraction method for measuring soil microbial biomass C. Soil Biol Biochem **19:**703-707.

2. **Brookes PC, Landman A, Pruden G, Jenkinson DS.** 1985. Chloroform fumigation and the release of soil nitrogen: A rapid direct extraction method to measure microbial biomass nitrogen in soil. Soil Biol Biochem **17:**837-842.
